# Characterization and In Vitro and In Vivo Assessment of a Novel Cellulose Acetate-Coated Mg-Based Alloy for Orthopedic Applications

**DOI:** 10.3390/ma10070686

**Published:** 2017-06-22

**Authors:** Patricia Neacsu, Adela Ioana Staras, Stefan Ioan Voicu, Iuliana Ionascu, Teodoru Soare, Seralp Uzun, Vasile Danut Cojocaru, Andreea Madalina Pandele, Sorin Mihai Croitoru, Florin Miculescu, Cosmin Mihai Cotrut, Ioan Dan, Anisoara Cimpean

**Affiliations:** 1 Department of Biochemistry and Molecular Biology, University of Bucharest, 91-95 Spl. Independentei, 050095 Bucharest, Romania; neacsu.patricia88@gmail.com (P.N.); adela_staras@yahoo.com (A.I.S.); 2 Department of Analytical Chemistry and Environmental Engineering, Politehnica University of Bucharest, 313 Spl. Independentei, 060042 Bucharest, Romania; stefan.voicu@upb.ro (S.I.V.); pandele.m.a@gmail.com (A.M.P.); 3 Department of Clinical Sciences, University of Agricultural Sciences and Veterinary Medicine, 105 Spl. Independentei, 050097 Bucharest, Romania; driulianaionascu10@gmail.com (I.I.); seralp.uzun@gmail.com (S.U.); 4 Pathology Department, University of Agricultural Sciences and Veterinary Medicine, 105 Spl. Independentei, 050097 Bucharest, Romania; teodoru.soare@gmail.com; 5 Materials Processing Department, Politehnica University of Bucharest, 313 Spl. Independentei, 060042 Bucharest, Romania; dan.cojocaru@mdef.pub.ro; 6 Machines and Manufacturing Systems Department, Politehnica University of Bucharest, 313 Spl. Independentei, 060042 Bucharest, Romania; sorin.croitoru@gmail.com; 7 Department of Metallic Materials Science, Physical Metallurgy, Politehnica University of Bucharest, 313 Spl. Independentei, 060042 Bucharest, Romania; florin.miculescu@upb.ro (F.M.); cosmin.cotrut@upb.ro (C.M.C.); 8 Experimental Physics Department, National Research Tomsk Polytechnic University, Lenin Avenue 43, 634050 Tomsk, Russia; 9 SC R&D Consulting and Services SRL, 45 Maria Ghiculeasa, 023761 Bucharest, Romania; ioan_dan@rd-consultanta.ro

**Keywords:** magnesium alloy, cellulose acetate coating, corrosion, osteoblast, in vivo behavior

## Abstract

Despite their good biocompatibility and adequate mechanical behavior, the main limitation of Mg alloys might be their high degradation rates in a physiological environment. In this study, a novel Mg-based alloy exhibiting an elastic modulus E = 42 GPa, Mg-1Ca-0.2Mn-0.6Zr, was synthesized and thermo-mechanically processed. In order to improve its performance as a temporary bone implant, a coating based on cellulose acetate (CA) was realized by using the dipping method. The formation of the polymer coating was demonstrated by FT-IR, XPS, SEM and corrosion behavior comparative analyses of both uncoated and CA-coated alloys. The potentiodynamic polarization test revealed that the CA coating significantly improved the corrosion resistance of the Mg alloy. Using a series of in vitro and in vivo experiments, the biocompatibility of both groups of biomaterials was assessed. In vitro experiments demonstrated that the media containing their extracts showed good cytocompatibility on MC3T3-E1 pre-osteoblasts in terms of cell adhesion and spreading, viability, proliferation and osteogenic differentiation. In vivo studies conducted in rats revealed that the intramedullary coated implant for fixation of femur fracture was more efficient in inducing bone regeneration than the uncoated one. In this manner, the present study suggests that the CA-coated Mg-based alloy holds promise for orthopedic aplications.

## 1. Introduction

Metallic magnesium (Mg) has attracted an increased interest for research and clinical applications due to its good biocompatibility, mechanical properties similar to natural bone, necessity in metabolic processes of the human body and capacity to degrade completely in the body environment. In spite of the huge potential of Mg and its alloys as bio-degradable implants, the major limitations of these materials are rapid and uncontrolled dissolution in the physiological environment accompanied by a rapid release of hydrogen bubbles. Pure Mg was implanted in a human body for the first time in the 1940s but later on it was abandoned because of the finding that the mechanical integrity of Mg was maintained only for 6–8 weeks while hydrogen gas was accumulated during the corrosion process [1,2].

In recent years, Mg-based biomaterials have regained attention for biomedical applications as promising potential candidates for the orthopedic field. In order to delay the corrosion rate of Mg and to improve its biological behavior, different modification methods, such as alloying and various surface coatings, have been introduced. For example, it was pointed out that Ca, Mn, Zn and Zr could be suitable alloying candidates because they are tolerated in the human body and can also delay biodegradation. The presence of these alloying elements can significantly improve the physical and mechanical properties of metallic alloys by refining the structure, improving the corrosion resistance, improving the mechanical strength by forming intermetallic phases and, also, by improving machinability. Ca, an essential element that can be metabolized in the human body, may exhibit anticarcinogenic properties and should be the first choice to be introduced into Mg-based alloys for biomedical implants. Moreover, this chemical element improves thermal and mechanical properties and refines grains in the structure [3]. Manganese is added to many Mg-based alloys to improve corrosion resistance and reduce the harmful effects of impurities [3]. Zr is a potential grain-refining agent for Mg-based alloys, helping to avoid heterogeneous nucleation and to obtain a significantly refined microstructure [4]. As a result, both mechanical strength and elongation of Zr-containing Mg alloys are much higher compared to Zr-free alloys. Also, Zr reduces the magnitude of the alloy degradation rate. Studies have shown that Mg-Ca, Mg-Zn and Mg-Mn-Zn alloys demonstrated good in vitro and in vivo biocompatibility and enhanced corrosion resistance, dissolving progressively within the bone tissue [5,6,7]. Likewise, surface modifications through applying different coatings on Mg or Mg alloys (such as hydroxyapatite (HA: Ca_10_(PO_4_)_6_(OH)_2_) on Mg-Zn [8,9], HA–chitosan on AZ31 [10,11], bioglass [12] or β-TCP on AZ31 [13,14], etc.) proved to efficiently slow down the degradation process of Mg-based biomaterials and to diminish the hydrogen evolution. In this context, the aim of the present study was to synthesize a cellulose acetate (CA) coating on a novel Mg-based alloy, namely Mg-1Ca-0.2Mn-0.6Zr (wt %), obtained by melting in a stir casting furnace. CA is an important and commonly used ester of cellulose with the advantages of being biocompatible and bioresorbable and is also widely accessible and cheaper than other potential polymers for metallic implant coatings, like polylactic acid or polycaprolactone. Moreover, CA membranes have been proved to display relatively low permeability of hydrogen gas [15,16] and a rejection rate for Mg^2+^ up to 99% [17]. Recent studies have shown that membranes containing bacterial cellulose could represent appropriate materials for tissue engineering purposes, allowing cell adhesion, viability and the expression of specific markers such as alkaline phosphatase (ALP), octamer-binding transcription factor 4 (OCT-4) and stage-specific embryonic antigen-4 (SSEA-4) [18]. Other studies that investigated the use of CA membranes as coatings of metallic implants showed that these materials were able to promote osteoblast proliferation, inducing bone growth around the implant [19,20]. A recent study introduced, for the first time, the use of CA-based membranes for controlling the dissolution of Mg [21]. It was demonstrated that CA-based membranes are able to control Mg dissolution and to regulate the associated pH increase. However, there are no data regarding the effects of Mg alloys coated with CA on the in vitro cellular response or in vivo osseointegration.

To better understand the impact of a novel material within the human body, extensive in vitro and in vivo investigations are required prior to clinical testing. It is generally accepted that the bone healing process is estimated at 4 to 12 weeks depending on the anatomical location of the bone. Therefore, it is desirable for Mg to maintain its mechanical properties over a period of 12 to 18 weeks, until the bone tissue regenerates [22]. While some in vivo studies have demonstrated long-term biocompatibility and good bone attachment to Mg-based implants from 9 to 18 weeks after implantation [6,23,24], in other cases, gaps could still be noticed at the bone-implant interface after 14 weeks of implantation even though the material showed a mild degradation rate [24]. These inconsistent results lead to the necessity of further experimentation in order to improve the bio-functionality of Mg-based materials.

Thus, in this study, in vitro biocompatibility and long-term in vivo osseointegration potential of a novel uncoated and CA-coated Mg alloy were evaluated to better understand the biological responses to Mg-based biomaterials. In vitro, both groups of Mg-based biomaterials had no cytotoxic effects against MC3T3-E1 pre-osteoblasts and supported cell adhesion, proliferation and differentiation. In vivo, bone regeneration was present in both CA-coated and uncoated implant groups, but in the second one, the regeneration was mainly represented by scar formation, suggesting that CA-coated Mg alloy possesses advantages as a bone implant over the uncoated alloy.

## 2. Materials and Methods

### 2.1. Materials Synthesis and Characterization

#### 2.1.1. Alloy Synthesis, Thermo-Mechanical Processing, Microstructural and Mechanical Characterization

The Mg-1Ca-0.2Mn-0.6Zr (wt %) alloy was produced starting from high-purity elemental components, in a stir casting furnace under an argon protective atmosphere. The melting temperature was 745 °C, and the melt was cast in Ø 25 mm × 100 mm ingots inside the melting furnace chamber.

After casting and turning machining (to remove the ingots outer layer), the ingots were hot extruded, at 400 °C, from Ø 20 mm to Ø 16 mm in a single step, with a total deformation degree of 36 %. In order to remove the internal stress from the extruded bar, a tempering heat treatment, at 180 °C for 7 min, was performed. From the tempered extruded bar, disc samples (diameter: 16 mm, thickness: 2 mm) were cut to be used in further experiments. Then, all samples were cleaned by washing in 70% ethanol (three 15 min-washes) followed by three 15-min washes in sterile milliQ water.

The samples used in the alloy’s microstructural analysis were metallographically prepared by the following procedure. All samples were cold mounted, using Buehler EpoxiCure resin, in Ø 30 mounts. The mounts were ground to 1000-grit using SiC paper pads and polished with 6 μm to 1 μm using Buehler MetaDi Oil polycrystalline diamond suspensions, followed by super-polishing with 0.05 µm Buehler Master Polish suspension. The polishing steps were executed on Buehler TexMet C polishing pads, while the super-polishing was performed on a Buehler ChemoMet polishing pad. After polishing, the prepared samples were etched by immersion for 30–60 s in a mixture of 100 mL ethanol +10 mL distilled water +5 mL acetic acid +6 g picric acid. The polishing and etching steps were followed by ultrasonic cleaning of the samples, for 5 min, in ethanol.

The microstructural analysis was performed using a Tescan Vega II-XMU scanning electron microscope (Tescan, Brno, Czech Republic). The mechanical characterization was performed on “dog-bone” samples, with 3 mm calibrated width, 1 mm calibrated thickness and 10 mm calibrated length, in tensile testing. The samples were cut from the tempered extruded bar along the extrusion direction. The mechanical characterization was performed using a micro-mechanical testing module DEBEN MicroTest 2000N (Deben, Woolpit, UK) at 0.4 mm/min testing speed.

#### 2.1.2. Coating Formulation

For alloys coating with CA, a solution of polymer in *N,N*’–dimethylformamide (DMF) was used. The solution was prepared by dissolving under vigorous stirring the polymer (Cellulose Acetate, 30% acetylation degree, Sigma-Aldrich, St. Louis, MO, USA) in DMF (Merck, analytic purity, Darmstadt, Germany) at a concentration of 12 wt % For alloy coating, the disc samples were dipped in polymer solution, and the solvent was evaporated at 45 °C for 5 days in order to remove all traces of the solvent. The operation was repeated three times until a uniform coating was achieved. After synthesis, the coated alloys were washed with ethanol (Riedel de Haen, analytical purity, Seelze, Germany).

#### 2.1.3. Biomaterials Characterization

Uncoated and CA-coated Mg-based alloy discs were morphologically characterized by Scanning Electron Microscopy (SEM) using a Philips XL 30 Instrument (Phillips, Eindhoven, The Netherland). Fourier Transform Infrared Spectroscopy (FT-IR) was performed using a Brucker Vertex 70 instrument (Bruker, Billerica, MA, USA) with ZnSe ATR annex, being recorded as a media of 32 measurements in a 550–4000 cm^−1^ range with a resolution of 1 cm^−1^. X-ray photoelectron spectroscopy (XPS) was carried out using an XPS−K ALPHA spectrophotometer (Thermo Scientific, Waltham, MA, USA).

The degradation behavior was determined with the Tafel plot electrochemical technique. This technique consists of linear polarization plotting curves involving the following steps: open circuit potential measurements for 1 h; potentiodynamic polarization plotting curves at ±250 mV vs. OCP (open circuit potential), with a scan rate of 1 mV/s. The experiments were made in simulated body fluid (SBF) solution at 37 ± 0.5 °C using a PARSTAT 4000 Potentiostat/Galvanostat (Princeton Applied Research—AMETEK, Oak Ridge, TN, USA) equipped with a glass with double wall (heating jacket), a saturated calomel electrode (SCE)—reference electrode, a platinum electrode—recording electrode and the working electrode, which consisted of coated and uncoated Mg based-alloy samples. The SBF was prepared according to Kokubo and Takadama [25], using commercially available reagents purchased from Sigma-Aldrich (Taufkirchen, Germany) and ultrapure water. All electrochemical tests were performed according to the ASTM G59-97 (reapproved 2014) standard [26].

### 2.2. In Vitro Cellular Response

#### 2.2.1. Preparation of Extracts of the Biomaterials

The extracts of both groups of biomaterials were prepared according to ISO 10993-12 standards [27]. Prior to the extraction procedure, the sample discs were sterilized under ultraviolet (UV) light overnight. Then, the specimens were immersed in Dulbecco's modified Eagle’s medium (DMEM), at a ratio of the surface area to the volume of the extraction medium of 3 cm^2^/mL and maintained at 37 °C for 24 h. The supernatants were collected, and the obtained extracts were 8x diluted with the appropriate culture medium and were further used in the cell-based assays.

#### 2.2.2. Cell Culture

Considering the potential use of Mg-based alloys in the orthopedic field, the pre-osteoblast MC3T3-E1 Subclone 4 cell line (American Type Culture Collection) was used in this study. The cells were grown in DMEM supplemented with 10% heat-inactivated fetal bovine serum and 1% penicillin/streptomycin in a humidified atmosphere of 5% CO_2_ at 37 °C. The medium was changed every 3 days during the incubation period. For further indirect contact experiments, MC3T3-E1 cells were trypsinized at about 80% confluence and seeded on 12-well-plates at an initial density of 1.5 × 10^4^ cells/cm^2^ in the corresponding extraction media for performing the experiments of cell attachment and spreading, morphology, viability and proliferation. In order to assess the pre-osteoblast differentiation, an initial cell density of 4 × 10^4^ cells/cm^2^ was used. These studies were conducted under two experimental conditions, namely, in the presence of the extraction media without (−OM) and with (+OM) a supplement of osteoinductive factors such as ascorbic acid (50 μg/mL) and β-glycerophosphate (5 mM).

#### 2.2.3. Quantitative and Qualitative Assessment of Cellular Survival and Cell Proliferation

In order to demonstrate the eventual cytotoxicity of the extraction media, the cellular survival was assessed by combining a quantitative method, namely MTT (3-(4,5-dimethylthiazol-2-yl)-2,5-diphenyltetrazolium bromide) assay with a qualitative one (calcein acetoxymethyl ester (AM)/ethidium homodimer-1 (EthD-1) cell staining). This assay was performed after 1, 3 and 5 days of culture as previously described [28]. The amount of formazan produced by metabolically active viable cells was recorded at 550 nm wavelength using a microplate reader (Thermo Scientific Appliskan, Vantaa, Finland).

At the same time points, a LIVE/DEAD viability/cytotoxicity assay was performed in accordance with the previously reported protocol [29]. Images of representative microscopic fields were taken under an Olympus IX71 inverted fluorescence microscope and captured using a Cell F Image acquiring system, which revealed the presence of living cells (bright green fluorecence) and dead cells (red fluorescence).

#### 2.2.4. Fluorescence Microscopic Evaluation of Cell Adhesion and Morphology

To investigate the effects of the extraction medium on the cell adhesion and cell morphological features, MC3T3-E1 pre-osteoblasts were fixed with 4% paraformaldehyde for 30 min at 25 °C and then thoroughly washed with PBS (phosphate-buffered saline). Then, the plasma membrane was permeabilized using a solution containing 0.1% Triton X-100 and 2% bovine serum albumin (BSA) to allow the dyes to penetrate the cells and to block the non-specific binding sites. To visualize the actin microfilaments and vinculin, samples were incubated with Alexa Flour-488 phalloidin (Molecular Probes, Eugene, OR, USA) and anti-vinculin antibody (Santa Cruz Biotechnology, Dallas, TX, USA) followed by specific secondary antibody coupled with Alexa Fluor 546 Molecular Probes, Eugene, OR, USA), as previously described [29]. Finally, a 10 min incubation with 4′,6-diamidino-2-phenylindole (DAPI, Sigma-Aldrich, Steinheim, Germany) dye was performed in order to highlight the nuclei.

#### 2.2.5. Determination of Intracellular Alkaline Phosphatase Activity

Intracellular ALP activity was measured using a commercial kit (Alkaline Phosphatase Activity Colorimetric Assay Kit, BioVision, Milpitas, CA, USA) according to the manufacturer’s instructions. For this purpose, MC3T3-E1 cells grown for 7 and 14 days in the samples extraction media were lysed using a lysis buffer provided by the kit and then centrifuged in order to remove the cellular debris. For the reaction, 80 μL of supernatant containing the sample of interest was mixed with 50 μL of 5 mM p-nitrophenylphosphate (pNPP) and incubated for 60 min at 25 °C in the dark. Next, 20 μL of stop solution was added to each sample. The absorbance was measured at a wavelength of 405 nm using a microplate reader (Thermo Scientific Appliskan, Vantaa, Finland), and the corresponding values were related to a standard curve to determine the concentrations of the reaction product. To eliminate the variations due to the differences in protein amounts, the protein concentrations were previously measured for each sample using the Bradford reaction, and then the concentrations of p-nitrophenol were normalized to 1 mg/mL protein. Finally, ALP activity was calculated as follows: ALP activity (U/mL) = A/V/T, where A represents the amount of p-nitrophenol (pNP) expressed by the samples (in μmol), V is the volume of cell lysate used in reaction (in mL) and T is the reaction time (in min).

#### 2.2.6. Quantification of Osteopontin Secretion

Extracellular expression of osteopontin (OPN) was determined in the samples extraction media after 14 and 21 days of incubation. This study was performed using the enzyme-linked immunosorbent assay (ELISA) technique, according the manufacturer’s instructions (R&D Systems). Briefly, 50 μL of each sample was mixed with an Assay Diluent provided by the kit and incubated for 2 h at room temperature. After five washes with Wash Buffer solution, 100 μL OPN Conjugate was added to each well and then incubated for 2 h at room temperature. After an additional five washes, the samples were incubated with 100 μL Substrate Solution for 30 min protected from light. Finally, after the addition of 100 μL Stop Solution, the optical density of the compound was measured at 450 nm using a microplate reader (Thermo Scientific Appliskan, Vantaa, Finland).

#### 2.2.7. Quantitative Assessment of Extracellular Matrix Mineralization

The formation of extracellular calcium deposits by MC3T3-E1 pre-osteoblasts grown for 4 and 6 weeks in the extraction media under standard and osteogenic culture conditions was detected by Alizarin red staining. Briefly, at the end of the experimental period, the cells were washed twice with PBS and fixed with 10% paraformaldehyde for 20 min at 25 °C. After rinsing thoroughly with distilled water, 500 μL/well of Alizarin red solution was added and incubated for 60 min at room temperature. The dye was discarded, and the cell monolayer was washed with distilled water. To quantify the mineralization level, 100 μL of 5% perchloric acid was added to the cells and incubated for 10 min. Finally, the absorbance of the resulted suspensions was measured at 405 nm using a microplate reader (Thermo Scientific Appliskan, Vantaa, Finland).

### 2.3. In Vivo Animal Studies

#### 2.3.1. Implant Preparation and Surgery

Ingots of Mg-1Ca-0.2Mn-0.6Zr (wt %) with a diameter of 16 mm and a length of 50 mm were used to obtain the implants for in vivo experiments. Using a saw milling cutter 1 mm thick, several longitudinal plates 2.5 mm thick were cut, and, then rectangular longitudinal bars 2.5 mm × 2.5 mm were cut. These bars were turned to 2 mm diameter on a fine mechanics lathe with a normal lathe cutter made of High-Speed Steel (HSS). Turning was performed in successive steps: a rectangular bar was fixed into lathe’s chuck with a 3 mm output console, turned to 2 mm diameter and taken out with another 3 mm in the console, repeating the operation. After reaching 16 mm in length, the bar was cut off. From each rectangular bar, two 2 mm × 16 mm implants were obtained. The implants were washed in an ultrasonic bath with an anti-grease detergent solution (1:10 parts volume). Washed implants were rinsed in clean water, treated with water steam pressure and, finally, dried with air pressure.

Prior to surgery, all implants were sterilized upon UV light exposure overnight in a laminar flow hood and then packaged in sterile pouches. Male Albinos rats, 4 months old with a body weight of 0.250 kg were used for in vivo study. This study was approved by the Bioethics Committee of the University of Agronomic Sciences and Veterinary Medicine of Bucharest (Approval code: 06/04.04.2016). The rats were randomized into three groups of 4 animals each. In order to achieve the surgical approach, premedication was performed by intraperitoneally administering 0.25 mL per 100 g of body weight (BW) of solution consisting of ketamine 100 mg/mL (1.2 mL), Dexdomitor 0.5 mg/mL (0.8 mL) and 2 mL of saline. The maintenance of anaesthesia was achieved with 1–1.5% isoflurane on the mask. The surgery area was shaved, and the surface was disinfected with iodine solution. A 3 cm longitudinal incision of the skin was made on the anterior femoral region under sterile conditions. The connective tissue was poorly represented. Using a scissor blunt, dissection of the muscles was performed, and muscles detached easily from the surface of the femur. Thigh muscles were fixed using a lid retractor. The fracture of the femur was performed transversally in the middle third, and the implant was introduced intramedullary through the outbreak of the fracture. The implantations were made in both posterior legs (femur). In the right femur was introduced the uncoated Mg-based implant, and in the left femur, the coated one was implanted. Thigh muscles were then sutured with absorbable (PDS 3/0) in separate points and skin with non-absorbable (nylon 4/0) in surjet. After the surgery, antibiotic (Enroxil–10 mg/kg BW) and anti-inflammatory medicine (Metacam–0.2 mg/kg BW) were administered subcutaneously for six days. The surgical wound was healed per primam, without local treatment.

Radiographs were taken directly after surgery with the purpose of monitoring the callus formation and bone fracture healing process at 14, 30, 60, 90 and 180 days after the surgery. The fracture was radiologically highlighted, but the implants were radiolucent. No differences in the radiographic appearance of the femurs were marked. All the animals were kept under close observation over the experimental period (up to 180 days), during which time no mortality or morbidity in the animals was observed. At the end of each in vivo experimental period, the femurs were harvested by disarticulation. Prior to this, the rats received the same premedication as in the case of the surgical procedure for implantation. Euthanasia was performed with T61 (0.5 mL), administered intraperitoneally.

#### 2.3.2. Histological Examination

Immediately after euthanizing the animals, all the bone samples were fixed in 10 % buffered formaldehyde for a period of 48 h. After fixation, the specimens were decalcified in ethylenediaminetetraacetic acid (EDTA) until the bone structures were ready (soft) for trimming. Then, the specimens were embedded in paraffin wax. Sections (3–4 µm) were prepared and stained with haematoxylin and eosin (HE) for histological examination. The slides were observed using an Olympus BX41 microscope, and the images were captured by an Olympus DP25 Camera (Cell B software).

### 2.4. Statistical Analysis

The statistical correlation between samples was determined using one-way ANOVA software (Bonfferoni’s multiple comparison test). The mean ± SD was determined for each sample group in a given experiment, and differences with *p* ≤ 0.05 were considered statistically significant.

## 3. Results

### 3.1. Alloy’s Microstructural and Mechanical Characterization

**Figure 1:**
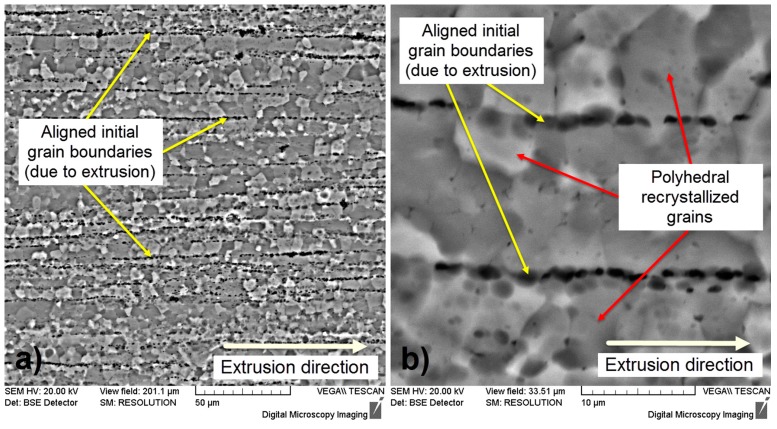
SEM-BSE (backscattered electrons) micrographs of Mg-1Ca-0.2Mn-0.6Zr alloy; (**a**) X750; (**b**) X3000.

Figure 1 illustrates specific microstructures of the Mg-1Ca-0.2Mn-0.6Zr alloy in a thermo-mechanical processed state. One can observe that the microstructure shows a fragmented and layered morphology, aligned with extrusion direction (Figure 1a). The layers are defined by the initial grain boundaries, prior to extrusion, due to the intense plastic deformation performed in a single step, with a total deformation degree of 36%. Also, one can observe that the layers contain polyhedral recrystallized grains due to the high deformation temperature, 400 °C, which assures the existence of dynamic recrystallization during deformation (Figure 1b).

**Figure 2:**
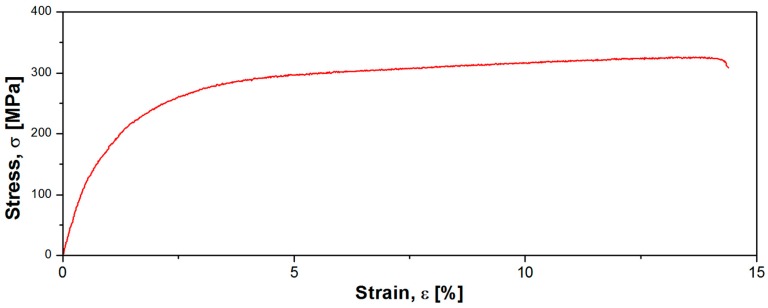
Strain-stress profile of the Mg-1Ca-0.2Mn-0.6Zr alloy in a thermo-mechanical processed state.

Figure 2 illustrates strain-stress profile of the Mg-1Ca-0.2Mn-0.6Zr alloy in a thermo-mechanical processed state. The mechanical characterization aimed to determine the mechanical properties of the developed alloy, expressed by 0.2 yield strength (σ_0.2_), ultimate tensile strength (σ_UTS_), elongation to fracture (ε_f_), and elastic modulus (E). Based on the strain-stress profile (Figure 2) one can compute the mechanical properties as follows: σ_0.2_ = 154.05 MPa, σ_UTS_ = 331.16 MPa, ε_f_ = 14.37% and E = 42.15 GPa.

If considering the elastic modulus, one can observe that the Mg-1Ca-0.2Mn-0.6Zr alloy exhibits an elastic modulus E = 42 GPa, which is very close to elastic modulus of human bone ~35 GPa [30,31], assuring “mechanical compatibility” with human bone if used as an osseous implantable material.

### 3.2. Characterization of Uncoated and Coated Mg-Based Alloy Samples

**Figure 3:**
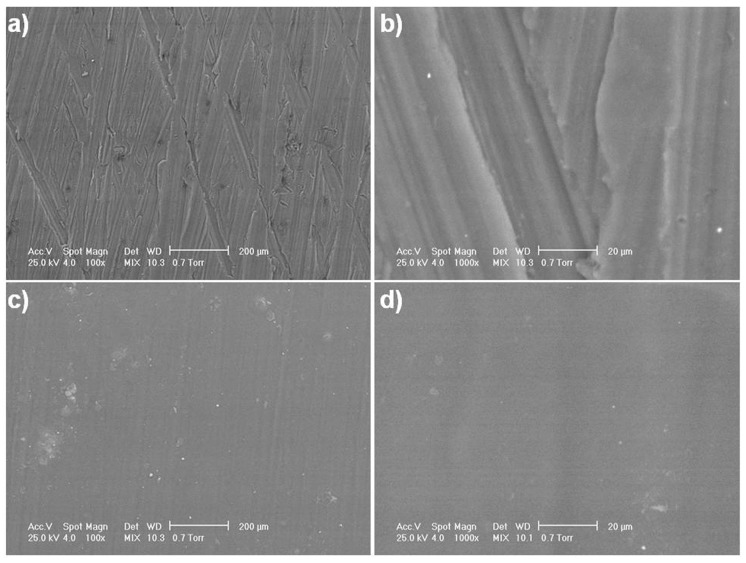

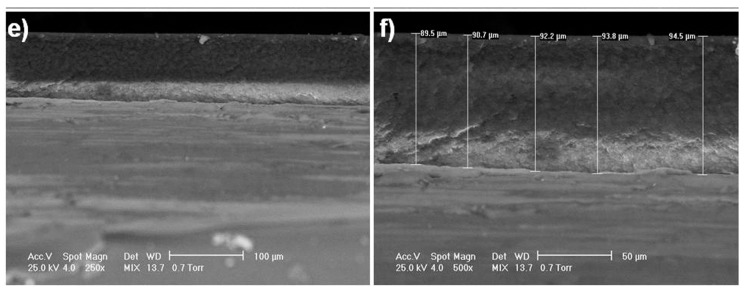
SEM micrographs of the uncoated and CA-coated Mg-1Ca-0.2Mn-0.6Zr alloy. Top view images of the uncoated (**a**,**b**) and CA-coated (**c**,**d**) alloy; Cross-section images of the CA-coated Mg alloy (**e**,**f**).

Top view SEM micrographs (Figure 3a–d) revealed surface morphology differences between the uncoated and coated alloy. On the surface of the uncoated alloy, micro-scratches from metallographic sample preparation can be observed on two directions (Figure 3a,b). Following the alloy coating, a smooth surface appears in SEM analysis due to the presence of the polymeric membrane (Figure 3c,d). By solvent evaporation, very compact polymeric films are synthesized with small-diameter pores, conferring a smooth character to the surface. Still, porosity exists due to the action of solvent molecules on the polymeric film during the evaporation process, which leads to the formation of channels. Cross-section SEM images of the coated Mg alloy (Figure 3e,f) show a homogenous CA film on the alloy surface. This polymeric film exhibits an average thickness of approximately 90 µm. The strength of the coating is indicated by its block uniformity.

**Figure 4:**
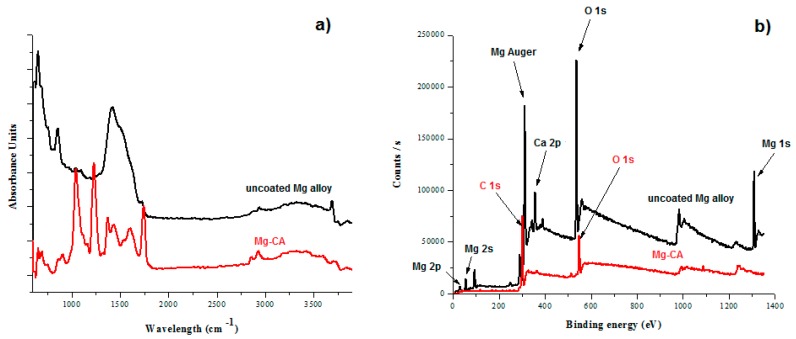
FT-IR (**a**) and XPS (**b**) spectra of the uncoated and coated Mg-based alloy.

From the FT-IR spectra (Figure 4), differences between studied materials can be observed, with a more complex spectrum in the case of the CA-coated alloy due to the presence of the polymer. A group of bands of different intensities can be observed in the 800−1750 cm^−1^ area [32], consisting of three strong peaks located at ~1100, ~1250, and ~1750 cm^−1^ assigned to asymmetric C–O–C (pyranose groups), C–O (COCH_3_ linked to the O originated from pyranose –OH), and C–O (ester) stretching vibrations, repsectively [33]. The peaks at ~1390 and ~900 cm^−1^ were assigned to CH_3_ and C–H in plane and out of plane bending vibrations, respectively [34]. Differences between FT-IR spectra of both analyzed biomaterials prove the presence of CA on the alloy surface.

XPS spectra (Figure 4) exhibit very distinct curves for the uncoated and coated alloy and can be correlated with the FT-IR analysis and SEM inspection results. In the case of the uncoated Mg-based alloy, there is a very distinct peak with high intensity at 320 eV attributed to Mg KLL Auger from the alloy. Other major Mg peaks are attributed to hybridization 2p (small intensity, at 30 eV), 2s (small intensity, at 98 eV) and 1s (medium intensity, at 1340 eV). The calcium from alloy appears at 350 eV (medium intensity) at 2p hybridization. Due to the small elemental complexity of the CA coating, the majority elements are carbon 1s hybridization (300 eV, medium intensity) and oxygen 1s hybridization at 540 eV.

**Figure 5:**
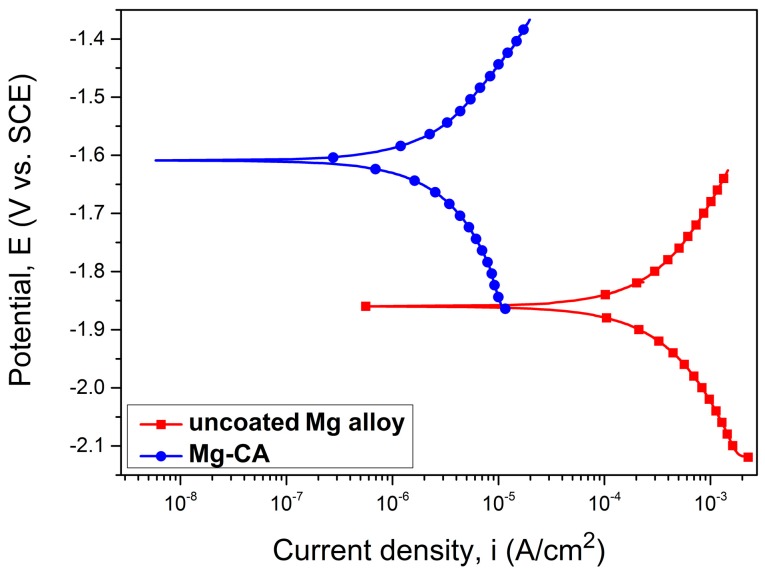
Tafel curves of the uncoated and coated Mg-based alloy.

The Tafel curves, corresponding to the uncoated and coated Mg-based alloy, in SBF medium, are shown in Figure 5.

The main electrochemical parameters are presented in Table 1. Degradation behavior of the analyzed specimens has been examined from different evaluation criteria. The higher electropositive corrosion potential (Ecorr) shows a better corrosion resistance. As can be observed from this table, the CA-coated Mg alloy has a higher electropositive corrosion potential value (−1.60 V). The corrosion current density (i_corr_) of the coated alloy is lower (4.88 μA·cm^−2^) than that of the uncoated specimens (497.96 μA·cm^−2^). Therefore, the corrosion current density of the Mg-CA sample is approx. 100 times smaller than i_corr_ recorded for the uncoated alloy, and consequently, it exhibits a higher corrosion resistance.

From the Tafel plots, considering the graphical extrapolation of the anodic and cathodic branches, the polarization resistance (Rp) was calculated using the Stern-Geary equation. Comparison of the Rp values showed that the CA-coated Mg alloy revealed a higher polarization resistance in SBF. The protective efficiency (Pe) was also obtained by the electrochemical method (Equation (1)), taking into account the corrosion current values of the CA-coated (i_corr___c_) and uncoated (_icorr_u_) Mg-based substrate [35].

(1) $$P_{e}=(1-\frac{i_{corr\_c}}{i_{corr\_u}})\times 100$$

It can be observed that Pe has a value of 99.02%, showing the protective character of the CA coating against degradation of the Mg-based alloy. Therefore, the potentiodynamic polarization test revealed that the CA coating significantly improved the corrosion resistance of the newly developed alloy.

### 3.3. In Vitro Behavior of Osteoblast-Like Cells

#### 3.3.1. Cell Viability/Proliferation and Morphological Features

**Figure 6:**
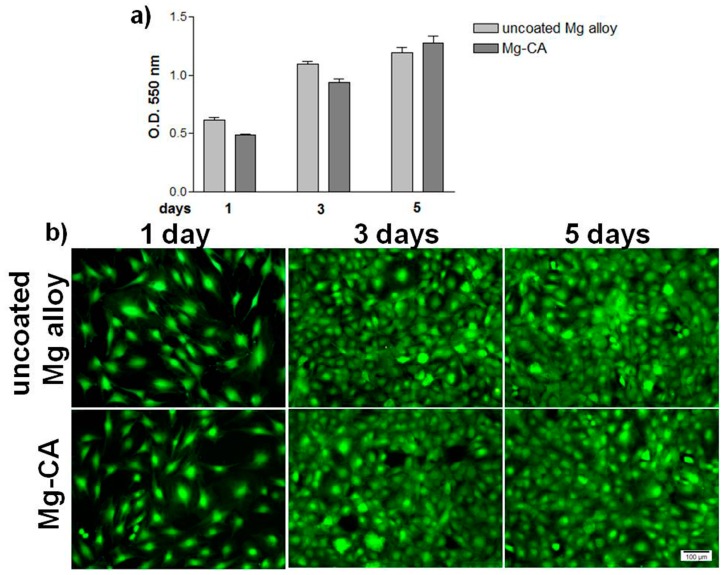
Cell viability/proliferation of MC3T3-E1 pre-osteoblasts grown in culture media containing the extracts of the uncoated and CA-coated Mg alloy. (**a**) MTT assay, formazan absorbance as a measure of cell proliferation. Results are presented as means ± SD (n = 3); (**b**) Fluorescent microscopy images of green-labeled living cells after perfoming the LIVE/DEAD cell viability assay.

The potential cytotoxic effects of the extraction media of uncoated and coated Mg-based alloy are shown in Figure 6. The ability of MC3T3-E1 pre-osteoblasts to proliferate and survive in these media was analyzed at 1, 3 and 5 days of culture. Cell proliferation was tested by MTT assay, which is based on the direct correlation between the activity of mitochondrial dehydrogenases of living cells and the number of cells attached to the substrate. As shown in Figure 6a, MC3T3-E1 cells showed a time-dependent increase in optical density (O.D.) in the case of both types of samples. In addition, the O.D. values did not show statistically significant differences between the cells grown in the two extraction media, although a slight increase is noted for the uncoated Mg alloy after 1 and 3 days of culture. These observations are supported by the results of the LIVE/DEAD test (Figure 6b). The fluorescent images reveal that the extraction media of both the uncoated and coated Mg-based alloy did not impair the viability of MC3T3-E1 pre-osteoblasts throughout the entire observation period. Importantly, no red-labeled dead cells were observed at the studied time points. Moreover, the results of this study showed a progressive increase in the number of viable osteoblasts over the experimental period of 5 days

**Figure 7:**
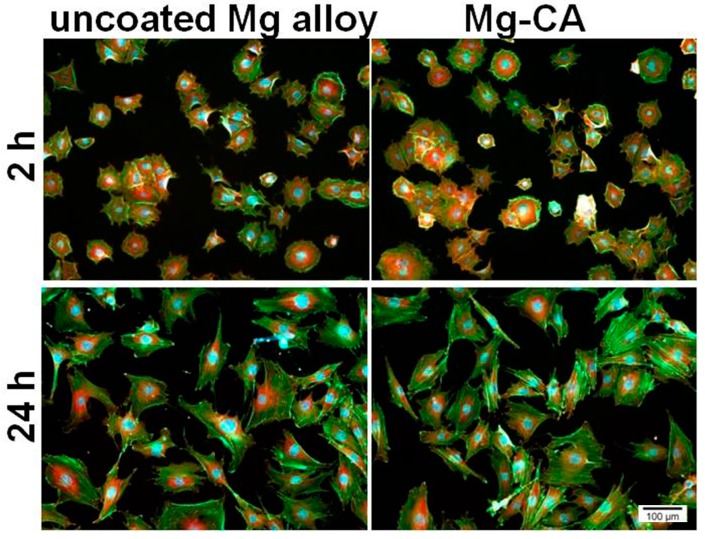
Fluorescent micrographs of MC3T3-E1 pre-osteoblasts grown in culture media containing the extracts of the uncoated and CA-coated Mg alloy. The cells were stained to detect actin (green) and vinculin (red). The nuclei are stained in blue with DAPI.

The ability of MC3T3-E1 cells to adhere and grow in the presence of the sample extraction media is shown in Figure 7. Both cell attachment capacity (at 2 h post-seeding) and cytoskeleton organization (at 24 h post-seeding) were studied by double labeling of actin and vinculin. Fluorescence microscopy images demonstrate the ability of cells to adhere to the culture substrate irrespective of the environment in which they were grown. Thereby, after 2 h of incubation, an intracellular distribution of vinculin, mainly around the nucleus, and the localization of actin predominantly at the periphery of the cells can be observed. Furthermore, after 24 h of culture, MC3T3-E1 cells maintained their interactions with the substrate and displayed a progressive increase in their size. As can be seen in the figure, the osteoblasts showed a typical morphology with elongated shape and stress fibers were arranged in well-defined parallel bundles along the cellular axis. As regards the cell spreading and distribution, no significant differences in the cell density between the two tested extraction media were observed at any time point. Therefore, the particles released from the uncoated and coated Mg alloy did not affect the adhesion, morphology and spreading of MC3T3-E1 pre-osteoblasts.

#### 3.3.2. The Function of MC3T3-E1 Pre-Osteoblasts

**Figure 8:**
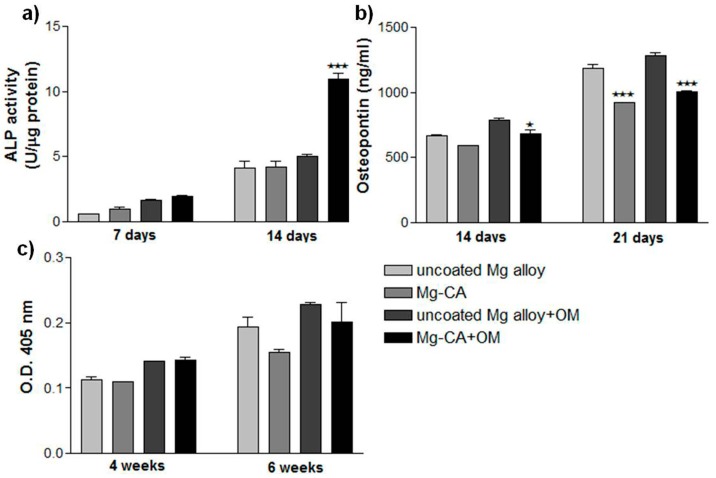
Osteoblastic differentiation of MC3T3-E1 cells cultured in the extraction media of the uncoated and CA-coated Mg alloy. (**a**) The levels of ALP activity (⋆⋆⋆ *p* < 0.001 vs. uncoated Mg alloy extraction media + OM); (**b**) The concentrations of osteopontin secreted (⋆ *p* < 0.05 vs. uncoated Mg alloy extraction media − OM; ⋆⋆⋆ *p* < 0.001 vs. uncoated Mg alloy extraction media + OM); (**c**) Quantitative colorimetric analysis of extracellular matrix mineralization. Results are presented as means ± SD (n = 3). +OM, with osteoinductive medium; −OM, without osteoinductive medium.

As an indicator of changes in the differentiation behavior of the bone-forming cells caused by the extraction media of both the uncoated and CA-coated Mg alloy, the intracellular ALP activity was measured after 7 and 14 days of cell incubation (Figure 8a). As shown in the figure, an increase in the activity of this enzyme was noticed over the culture period under both experimental conditions. Moreover, the addition of osteoinductive extraction medium led to a slight increase in the expression levels of ALP activity at both 7- and 14-day time points. The osteogenic differentiating MC3T3-E1 cells showed a similar pattern of ALP activity under both standard and osteoinductive experimental conditions after 7 days of culture. This effect was also exhibited by cells grown for 14 days in the absence of osteogenic stimulation. However, under osteogenic-inducing conditions, at this time point, a significant increase in ALP activity could be noted for the pre-osteoblasts incubated in the extraction media corresponding to the CA-coated Mg alloy as compared with the uncoated alloy.

To further assess the osteoblastic differentiation potential of MC3T3-E1 pre-osteoblasts, the concentration of osteopontin secreted in response to the compounds released from Mg-based biomaterials was studied by ELISA technique after 14 and 21 days of cell incubation (Figure 8b). The obtained results indicate a time-dependent increase in osteopontin secretion over the culture period under both experimental conditions. It is worth mentioning that under any culture condition, both materials’ extraction media exhibited the ability to induce the synthesis of this bone matrix protein and its extracellular release. However, a stronger effect in inducing osteopontin secretion was exerted by the extraction medium of the uncoated alloy as compared to the extraction medium of CA-Mg alloy, especially after 21 days.

The ability of these extraction media to induce extracellular matrix mineralization by murine MC3T3-E1 pre-osteoblasts was investigated by Alizarin red staining after 4 and 6 weeks of incubation. At the studied time points, a diffuse mineral deposition was noticed, denoting that the early stage of bone mineralization was in progress. The extent of mineralization corresponding to each sample was spectrophotometrically quantified, and the results obtained are presented in Figure 8c. It can be noticed that both analyzed extraction media induced early extracellular matrix mineralization with no statistically significant differences (*p* > 0.05) between them. However, it is worth mentioning that after 6 weeks of culture, a slightly lower degree of mineralization was induced by the extraction media corresponding to the Mg-CA sample as compared to the uncoated Mg alloy (with a decrease of ~20% under standard culture conditions and ~11.5% under osteoinductive conditions).

### 3.4. In Vivo Biocompatibility of the Mg-Based Biomaterials

Implantation in the femur of the Mg-based implants was carried out without complications due to the protocol of anesthesia and the surgical approach. The daily clinical monitoring and radiographs made at 14, 30, 60 and 180 days showed the tissue tolerance of the two types of implants. Important to note is that the alloy is radiolucent. From the radiological aspect, the healing process showed no differences between the two types of biomaterials. Furthermore, visible hydrogen bubbles could not be identified in the tissue surrounding both implant types.

**Figure 9:**
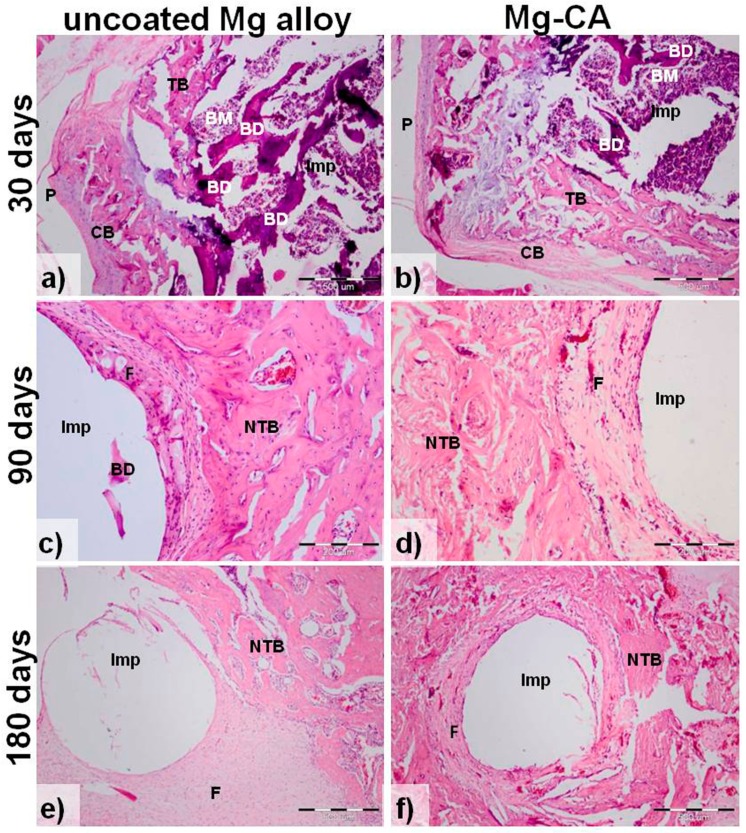
Hematoxylin and eosin (HE) staining of bone sections implanted with the uncoated (**a**,**c**,**e**) and CA-coated (**b**,**d**,**f**) Mg-1Ca-0.2Mn-0.6Zr alloy at: 30-, 90- and 180- post-implantation days. Imp, intra-medullary implant site; BM, bone marrow; BD, bone destruction; TB, trabecular bone; CB, compact bone; P, periosteum; NTB, newly formed trabecular bone; F- fibrosis. The white hole in (c–f) resulted by removing the residual Mg-based implant. Scale bars: 500 µm (a,b,e,f); 200 µm (c,d).

Generally, the histological sections from all bones implanted with the uncoated (Figure 9a,c,e) and CA-coated (Figure 9b,d,f) Mg-based alloy showed structural alteration (Figure 9a,b) with fractured bone lamellae, increased basophilia and lost cellular details. Also, new bone formation and peri-implant fibrosis (Figure 9c–f) are observed. In comparison with the uncoated implant group, the subjects with CA-coated implants showed less bone destruction and mild to moderate fibrosis (Figure 9d,f). Dense fibrous peri-implant tissue was mainly seen in animals implanted with uncoated Mg-based alloy, at 90 days (Figure 9c) and 180 post-implantation days (Figure 9e). Bone regeneration was present in both CA-coated and uncoated implant groups, but in the second one, the regeneration was mainly represented by scar formation. It should be mentioned that the void spaces resulted from removing the residual Mg-based implants are not that visible after 30 days of implantation (Figure 9a,b) because the connective tissue is still soft, not consolidated; the fibers are very thin and not oriented. Instead, at 90 and 180 days post-implantation (Figure 9c–f), the collagen fibers are oriented and maintain the implant space.

## 4. Discussion

Metallic Mg and its alloys have many unique qualities making them suitable for medical applications as potential load-bearing orthopedic implant materials. Despite their good biocompatibility and superior mechanical properties, the major concerns of Mg-based biomaterials are their rapid and non-uniform degradation. In this study, a CA coating formulation was proposed for corrosion protection of the Mg-1Ca-0.2Mn-0.6Zr alloy. This coating was previously investigated showing increased stability in physiological pH solution and a good osteoblast response in terms of cell adhesion, viability and proliferation [32]. Herein, are presented the synthesis method and the coating procedure with CA of the Mg-1Ca-0.2Mn-0.6Zr alloy as well as the results of the advanced physicochemical characterization and corrosion behavior of obtained biomaterials. Moreover, this study presents comparative data regarding their initial in vitro biocompatibility and in vivo osseointegration potential.

Cellulose is a syndiotactic homopolymer composed of D-glucopyranose units connected through β-(1-4)-glycosidic bonds, being the most common organic material in nature, with an abundance of about 5 × 10^11^ tons generated annually in the biosphere. It exhibits excellent properties, such as good mechanical strength, good biocompatibility and hydrophilicity, high sorption capacity and relatively good thermal resistance [36]. Due to the fact that cellulose is not soluble in usual solvents, being dissolved in highly toxic or difficult to remove mixtures, such as *N,N*-dimethyl acetamide/LiCl, the use of cellulose derivatives is generally preferred in practice. The most important cellulose derivatives are carboxymethyl cellulose, hydroxypropyl cellulose, hydroxyethyl cellulose, nitrocellulose and cellulose acetate, soluble in a wide range of common organic solvents [37]. The main reason for using CA as a coating material was its ability to hydrolyze and form nontoxic components such as free acetate and glucose. Our previously performed studies revealed a degradation rate of a CA membrane with approximately 45% of weight loss in aqueous solution at physiological pH, after 12 weeks [32]. Polymeric membranes can be obtained using a wide range of techniques, like polymer precipitation [38] using a nonsolvent for polymer, but totally miscible with polymer solvent or solvent evaporation [39]. In the first case, obtained membranes have pores in the micrometer range being suitable for different kinds of separations. By solvent evaporation, polymeric films are more compact, with pores in the nanometer range, and also provide a better protection of the developed Mg-based alloy. Furthermore, the precipitation of CA in the presence of a nonsolvent, which is water in this case, would be impossible due the corrosion character of this alloy. The repeated procedure for synthesize successive layers of the polymer coating allows a better protection of the Mg alloy against corrosion. It was also observed that the protective efficiency has a value of 99.02%, showing the protective character of the CA coating against degradation of the metallic substrate. Furthermore, the potentiodynamic polarization test revealed that the CA coating significantly improved the corrosion resistance of the Mg alloy. There have also been other studies showing the biodegradable behavior of coated Mg alloys. For instance, a composite HA-chitosan coating was deposited on AZ31 Mg alloy by aerosol deposition in order to improve the corrosion resistance and in vitro biocompatibility of the alloy [11]. The Ecorr values of the coated AZ31 Mg alloy immersed in SBF were much more positive than those of the uncoated alloy, and the coatings exhibited much lower Icorr values as compared to the uncoated AZ31 Mg alloy. These findings suggested that the corrosion resistance of the AZ31 Mg alloy was remarkably enhanced by coating with HA–chitosan composite material. In addition, the coated material showed a better MC3T3-E1 cell attachment. A very good coating was developed from CA and a polyelectrolyte, namely, poly(*N*,*N*-dimethylaminoethyl methacrylate (PDMAEMA), which consumes the hydrogen released from the Mg substrate [21]. Electrochemical measurements (linear sweep voltammograms, open-circuit potential, and polarization) showed that by altering the CA:PDMAEMA ratio, the dissolution rate of Mg can be controlled.

In vitro studies are often performed before biomaterial implantation in order to reveal their potential effects on the host cells. Cells are known to be very sensitive to fluctuations of the environment such as ion release, changes in the pH and hydrogen evolution [40]. Preliminary studies regarding the effects of the uncoated and CA-coated Mg alloy on the adhesion and viability of MC3T3-E1 pre-osteoblasts in direct contact with these substrates have shown that the cellular survival was strongly affected by changes of the materials’ surface due to the corrosion process. Witte et al. [41] have shown that the direct cell assay reduces cell viability more rapidly than the indirect cytotoxicity tests. Consequently, this study was performed using the extraction media prepared according to ISO 10993-12 standards. However, the corrosion process of Mg-based materials results in highly concentrated extracts with very high osmolality and several corrosion products [42]. Under in vivo conditions, these changes in the local environment are regulated by active transport processes of the human body [42]. Therefore, diluted extraction solutions were used in the experiments performed in this study. MC3T3-E1 pre-osteoblasts were exposed to the culture media containing the extracts of the uncoated and CA-coated Mg alloy, and two important particularities of the material-cell interaction were assessed, namely, cell survival/proliferation and functionality, in order to estimate the initial influence of the tested materials. The obtained results showed that the cells adhered and proliferated well in the extraction media of both analyzed biomaterials over the culture period, and slight differences were found between experimental conditions. Furthermore, no dead cells were observed, and viable pre-osteoblasts colonized the culture substrates, almost reaching confluence after 5 days of incubation. Fluorescence microscopic observation of the cytoskeleton after 24 h of culture revealed a healthy population of MC3T3-E1 pre-osteoblasts with well-organized actin microfilaments and focal adhesion structures. Given these results, it can be speculated that the initial cellular response was not altered by the compounds released in the extraction media. In the literature, Mg is described as an active element in the process of cell adhesion. Paul and Sharma [43] showed that cell attachment and spreading were significantly increased in the presence of Mg and Zn. Other studies indicate that the modification of biomaterials with Mg^2+^ resulted in increased adhesion of osteoblasts, and the substitution of tricalcium phosphate with Mg led to enhanced cell proliferation and collagenase synthesis [44,45]. In addition, recent studies suggest that Mg ions have the potential to enhance the cell response in the initial phase of the osseointegration process [46,47]. Moreover, Cifuentes et al. [48] have shown that by including small amounts of Mg particles into a poly-D-L-lactic acid (PDLLA) matrix, stimulation of fibronectin production, ALP activity and vascular endothelial growth factor secretion was induced in human bone marrow-derived mesenchymal stem cells as well as a reduction of the inflammatory response exhibited by human THP-1 macrophages.

Besides cell adhesion and proliferation, the capacity of materials to induce osteogenic differentiation is essential for the bone regeneration process. Osteoblasts have the ability to synthesize and secrete inorganic and organic constituents of the extracellular matrix. The matrix maturation process is accompanied by the expression of ALP and various proteins such as osteopontin, osteocalcin and bone sialoprotein [49]. Moreover, the cells undergoing matrix mineralization accumulate calcium in the form of intracellular deposits [50]. In this study, the long-term effects of the culture media containing the extracts of the uncoated and CA-coated Mg-alloy on the response of MC3T3-E1 pre-osteoblasts were investigated by assessing the ALP activity, OPN secretion and extracellular matrix mineralization. The obtained results showed that both extraction media have similar effects on ALP activity under standard culture conditions. However, under osteogenic-inducing conditions, the cells grown in the extraction medium of the CA-coated Mg alloy expressed increased levels of ALP activity compared to the cells grown in the extraction medium of the uncoated alloy. High levels of ALP activity demonstrate the ability of MC3T3-E1 cells to differentiate into mature osteoblasts. Similar results were observed in a recent study, which showed that the ALP activity of the bone mesenchymal stem cells in the extraction media of a Mg_2_SiO_2_-containing microarc oxidation (MAO) coated ZK60 Mg alloy was much higher than that of naked alloy [51]. Another important mediator of bone remodeling is OPN, which has been shown to act as a multifunctional protein. This study revealed that the exposure of MC3T3-E1 pre-osteoblasts to both extraction media induced the expression and secretion of OPN in the culture media. A significant decrease in this protein was noticed in the medium containing the extract of the CA-coated Mg alloy. OPN is an extracellular matrix protein involved in normal physiological processes, having different biological functions including the regulation of osteoblastic differentiation or bone remodeling process [52]. Phosphorylation of OPN has been demonstrated to play a role in the inhibition of biomineral formation and growth in vitro [53]. Moreover, Huang et al. [54] concluded that OPN is a negative regulator of proliferation and differentiation of MC3T3-E1 cells; overexpression of this protein causes decreases in expression of osteocalcin and bone sialoprotein and inhibits mineral deposition. Our findings suggest that reduced concentrations of OPN secreted by the cells maintained in the extraction media of the CA-coated Mg alloy may have a positive impact on further bone mineralization. However, at the studied time points, cells seemed to be in the early phase of mineralization as no individual mineral nodules were observed in culture. Overall, the results of in vitro experiments showed that the media containing the extracts of uncoated and CA-coated Mg alloy did not exhibit cytotoxic effects against MC3T3-E1 pre-osteoblasts. Furthermore, these findings reveal good osteoblastic cytocompatibility of both analyzed biomaterials in terms of cell adhesion, viability and proliferation, and promotion of osteogenic differentiation. Likewise, histological analysis of the implantation sites at 90 and 180 days after implantation shows that new bone tissue formed around both types of Mg-based implants, suggesting their good biocompatibility. However, fibrous tissue was found at the interface between the implants and the new bone tissue. Noteworthy, dense fibrous peri-implant tissue was mainly seen in animals implanted with the uncoated Mg-based alloy when regeneration was mainly represented by scar formation. Both implant types were degraded to only a limited extent throughout in vivo experimental periods. Therefore, in future work, we want to conduct these studies in the same animal model over a period of 9 and 12 months.

## 5. Conclusions

In this paper, we synthesized and studied a novel Mg-based alloy, Mg-1Ca-0.2Mn-0.6Zr (wt %) subjected to thermo-mechanical processing. Dip coating method in a solution of CA in *N,N*′-dimethylformamide followed by the evaporation of the solvent and polymer precipitation were applied in order to provide a protective layer. The formation of this layer was proved by FT-IR, XPS, SEM and corrosion behavior comparative analyses of both the uncoated and CA-coated alloy. In vitro and in vivo experiments were performed to investigate the biocompatibility of these Mg-based biomaterials. The results obtained proved good cytocompatibility of both groups of Mg-based biomaterials with respect to cell adhesion, viability and proliferation, and promotion of osteogenic differentiation. In vivo, bone regeneration was present in both implant groups, but the CA-coated implants showed less bone destruction and mild to moderate fibrosis while in the case of using uncoated Mg-based implants, the regeneration was mainly represented by scar formation. Therefore, the coated alloy was more efficient in inducing bone regeneration than the uncoated one.

## Figures and Tables

**Table 1:** Main electrochemical parameters of the uncoated and coated Mg-based alloy.

Sample	E_corr_ (V)	i_corr_ (µA/cm^2^)	−β_c_ (mV)	β_a_ (mV)	Rp (kΩxcm^2^)	P_e_ (%)
uncoated Mg alloy	−1.85	497.96	422.92	475.14	0.195	-
Mg-CA	−1.60	4.88	614.56	391.57	21.28	99.02
